# What place for pragmatic RCTs in intensive care?

**DOI:** 10.1186/s13054-026-06242-z

**Published:** 2026-08-12

**Authors:** Mervyn Singer, Jean-Louis Vincent

**Affiliations:** 1 Bloomsbury Institute of Intensive Care Medicine, UCL Respiratory, Division of Medicine, University College London, London, WC1E 6 BT UK; 2 Department of Intensive Care, Hopital Universitaire de Bruxelles (HUB), Université Libre de Bruxelles, Brussels, Belgium

## Abstract

Pragmatic trials test the effectiveness of an intervention within real-life clinical practice, prioritising applicability and generalisability. By contrast, explanatory trials evaluate the efficacy of an intervention under optimal conditions and in well-defined populations, aiming to minimise bias and confounding and to maximise the treatment effect. The majority of large-scale, multicentre academic ICU studies are overall pragmatic in nature but have repeatedly failed to demonstrate outcome differences against control populations. This relates in part to the marked heterogeneity of ICU populations, often with enrolment based on syndromic categories such as sepsis, acute respiratory distress syndrome, shock and acute kidney injury with significant inter-individual biological variability. Any beneficial treatment effects in specific, often unidentified, subsets are diluted or cancelled out by no effect or even harm in others. A second important factor underlying the lack of intervention effect is suboptimal performance of the trials, particularly poor protocol adherence, which undermines the likelihood of showing benefit, particularly when reliant on intention-to-treat analyses. In this article we discuss these issues and provide some suggestions for improvement.

**Supplementary Information:**

The online version contains supplementary material available at 10.1186/s13054-026-06242-z.

## Introduction

Critical care practice has evolved over decades with a reliance on consensus rather than hard evidence. This, in part, reflects the marked heterogeneity of the patient population, and the generally flawed assumption that a one size intervention will fit all. Other than demonstrating benefit from avoidance of harmful practices such as excessive ventilation, nutrition, fluid and sedation, most multicentre centre studies have failed to show a positive outcome to promote inclusion of the intervention into routine clinical management. Overall, in unselected populations, no ventilatory mode has been shown to be superior to another, no immunomodulatory intervention has been effective in sepsis, and no strategy of nutritional support can be recommended above others.

**Fig. 1:**
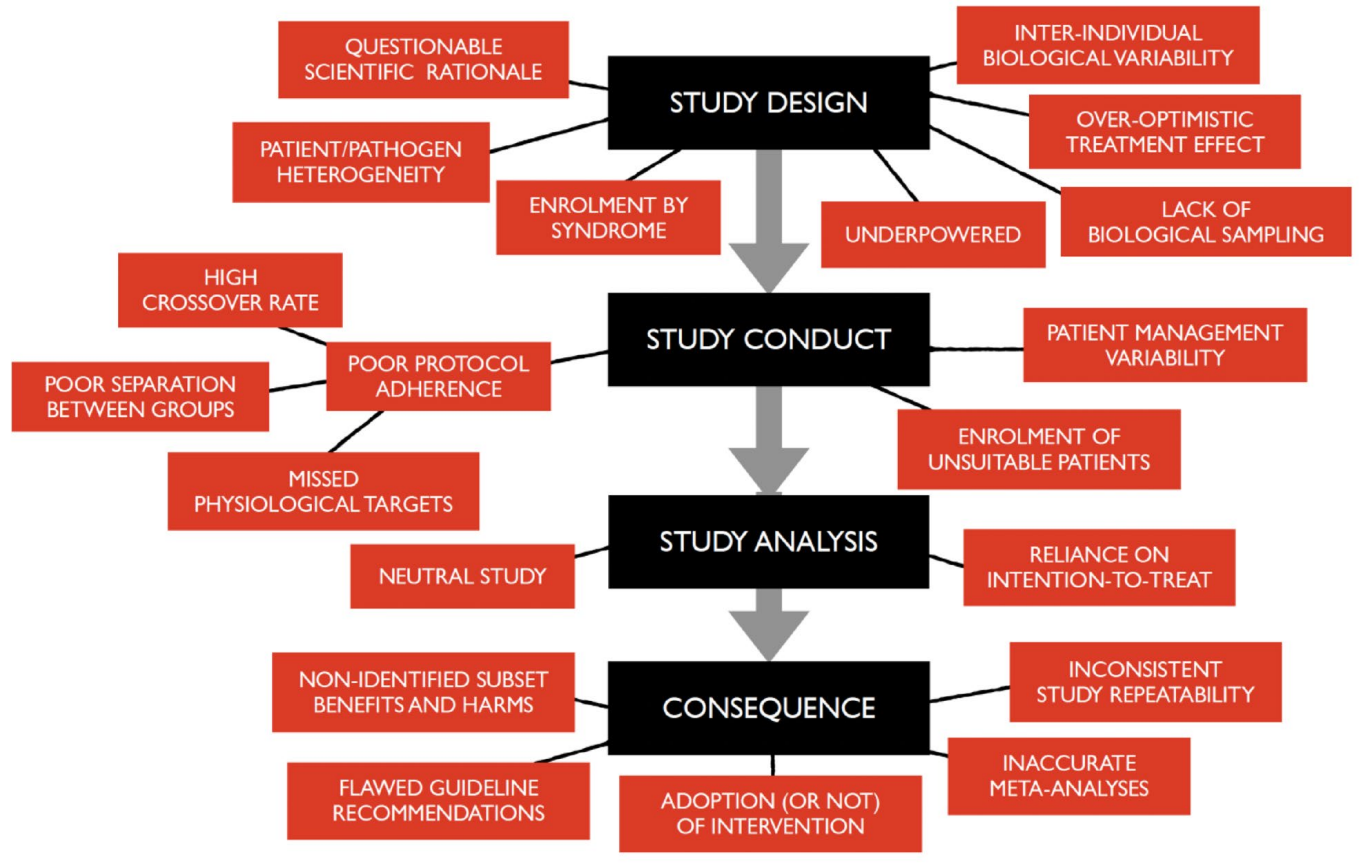
Examples of issues arising from flawed study design and conduct, with impact on analyses and downstream consequences on clinical management

In too many instances, flawed trial design and/or conduct have contributed to the above issues (Fig. 1). Unsuitable patients may be enrolled to meet recruitment targets. Examples include enrolling low-risk patients into studies where mortality was the primary outcome, or patients with minimal fluid requirements into fluid comparison studies. Conversely, sample size estimations are not infrequently based on an over-optimistic treatment effect to meet funding and/or recruitment limitations. Encouraging outcome trends have been observed in some underpowered studies, but failure to satisfy a p value < 0.05 consigned the intervention to a possibly undeserved rubbish skip.

Worryingly, a frequent inability to adhere to the study protocol results in physiological targets not being reached, and with inadequate separation between treatment and control groups. There is also a tendency to dismiss the intervention purely on the failure of the headline primary outcome (e.g. an arbitrary selection of a 28-day mortality difference) while disregarding pertinent subgroup analyses and important patient-centred secondary outcomes.

The pragmatic design and conduct of many of these studies can be held responsible for many of the neutral study outcomes. This article examines what is meant by a pragmatic trial, provides a few instances where design and/or trial performance have been suboptimal, and makes some recommendations to improve their application and execution.

### Explanatory *or* pragmatic?

In 1967 Schwartz and Lellouch coined the terms “explanatory” and “pragmatic” to distinguish between two fundamentally different approaches to clinical trials [1]. Explanatory trials are designed to evaluate an intervention’s *efficacy* under carefully controlled conditions, often utilising specific clinical or biological markers, and performed in well-defined populations. Their purpose is to determine whether - and how - an intervention works, with trial designs that attempt to minimise bias and confounding to maximise the effect of the intervention. Conversely, pragmatic trials test an intervention’s *effectiveness* within routine clinical practice. Such trials prioritise applicability and generalisability, addressing whether the intervention works in real-world settings. Pragmatic trials often include broader patient populations and measure a wider spectrum of outcomes, including patient-centred endpoints. To overcome the inherited heterogeneity, which can dilute the treatment effect, pragmatic trials have to be sufficiently large to increase power to detect small effects and produce reliable evidence. However, knowing a treatment is effective or not in a real-life setting does not provide a specific quantifiable answer for an individual patient, for instance, the effect of the intervention in an 80-year old male with hypertension and chronic renal failure.

In practice, randomised trials are neither fully explanatory *nor* pragmatic, but exist along a continuum. This concept was formalised by Thorpe and colleagues in 2009 through their development of the PRagmatic-Explanatory Continuum Indicator Summary (PRECIS), comprising 10 separate domains spanning key elements of trial design [2]. This was updated to PRECIS-2 in 2015 which incorporated nine domains – eligibility, criteria, recruitment, setting, organisation, flexibility (delivery), flexibility (adherence), follow-up, primary outcome, and primary analysis (Table 1) [3]. These domains are scored from 1 (very explanatory) to 5 (very pragmatic). As exemplified later, many domains within ICU studies, including trials that are ostensibly explanatory, score 4–5. The contribution of perhaps excessive pragmatism to the repeated failures of so many critical care RCTs to demonstrate an outcome benefit should not be underestimated.

**Table 1:** PRECIS-2 domains illustrating extremes of explanatory and pragmatic approaches. Adapted from Loudon et al. [3]

Domains	Explanation	Highly explanatory	Highly pragmatic
Eligibility criteria	To what degree are trial participants similar to those who would receive this intervention if it was part of usual care?	Tight inclusion and exclusion criteria	Include anyone with the condition of interest who would be likely to receive the intervention if provided in usual care
Recruitment	What additional effort is needed to recruit participants over and above what would be used in the usual care setting?	Extra resources to recruit patients	Recruit only from patients undergoing usual care, and/or from multiple centres to increase trial results’ generalisability
Setting	Where is the trial being performed?	Only performed in selected centres, e.g. specialist or academic Units	Perform in settings to which results are intended to be applied
Organisation	What expertise and resources are needed to deliver the intervention?	Use of dedicated research staff, with a level of experience working with the intervention, and with additional training to increase expertise	Apply intervention within usual organisation of care, using existing healthcare staff and resources.
Flexibility (delivery)	How should the intervention be delivered?	Intervention is highly-specified and protocol-driven. Timing of delivery is tightly defined to maximise effect, with restrictions on number and types of co-interventions, and specific directions for managing complications or side effects Measures in place to monitor compliance of those delivering the intervention in line with the protocol, and to address poor compliance	Details of implementation left to providers, i.e. the protocol is not rigidly prescriptive. No stipulation as to which other interventions are permitted, or how to deliver them
Flexibility (adherence)	What measures are in place to ensure participants adhere to the intervention?	Protocol lays out methods to monitor and ensure patient compliance. Applies to all intervention arms as well as the control arm (unless this is usual care).	Full flexibility in how patients engage with the intervention. No special measures to enforce engagement or compliance.
Follow-up	How closely are participants followed up?	More frequent follow-up visits than would typically occur under usual care, with more extensive data collection.	No more follow-up than would be the case in usual care. Minimal additional data such as outcome data obtained by other means (e.g. electronic medical records)
Primary outcome	How relevant is it to participants?	Use of: - a surrogate outcome (e.g. blood biomarker) that intervention is expected to impact upon; - a composite primary outcome with some elements being less important to patients or participants (e.g. 30-day mortality); - a central adjudication of the outcome or assessment that needs special training or tests - an outcome mainly important to providers, e.g. a physiological outcome considered useful in treatment planning and monitoring	Use of patient-centred outcomes, that may also be relevant to care commissioners who decide whether to implement the intervention based on the study results
Primary analysis	To what extent are all data included?		No special allowance made for non-adherence, practice variability, etc. This would typically be an intention-to-treat analysis using all available data

### Challenges presented by patients with critical illness

Unlike discrete disease entities such as lung cancer, thrombotic stroke or myocardial infarction - where anatomical, histological and genetic diagnostics guide directed interventions - trials in complex critically ill patients are particularly challenging. The overall ICU population is highly heterogeneous, varying widely in age, ethnicity, comorbidities, underlying pathology, time to presentation, reason for admission, and the number and severity of organ dysfunctions. Within trial designs, ICU patients are often conveniently selected through fulfilling syndromic labels such as ‘sepsis’, ‘acute respiratory distress syndrome’ (ARDS), ‘acute kidney injury’ (AKI) and ‘delirium’. Yet even within these categories, patient demographics, precipitating factors, illness severity and individual biological responses vary markedly. Inclusion criteria into trials are often inconsistent. For example, studies in patients with septic shock utilised multiple inclusion thresholds resulting in control group mortality rates ranging between 13.8% and 84.6% [4]. The lack of a definitive diagnosis can also confound. Pathogens are not identified in antibiotic trials in approximately 30–50% of cases. How many of these patients actually had an indisputable bacterial infection?

This patient heterogeneity is further compounded by significant iatrogenic influences from diverse pharmacological therapies and support devices, with often marked inconsistencies in management between ICUs. Potentially important confounding effects have been rarely captured within trials but do reflect real-life variability. For example, heavier use of sedation in some centres will result in an increased risk of haemodynamic instability with more use of fluid and vasopressors, prolonged duration of mechanical ventilation, immobility, acquired immunosuppression and other complications.

**Fig. 2:**
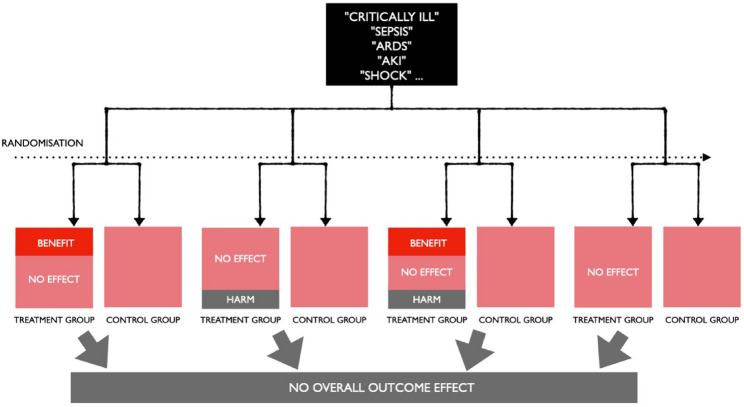
Possible scenarios leading to no overall effect in large multi-centre intervention studies. ARDS acute respiratory distress syndrome; AKI acute kidney injury

Taking into account all the above, it is hardly surprising that most pragmatic randomised controlled trials (RCTs) in critical care have failed to yield positive results. The same stricture also applies to explanatory RCTs that have incorporated too many pragmatic PRECIS domains. It is reasonable to assume that many tested interventions benefit some, harm others and have no effect in the remainder (Fig. 2). The failure to demonstrate statistically significant – and, more importantly, clinically meaningful - benefits across the overall study population often results in the intervention being deemed futile and often discarded. Yet this ‘neutral’ result disregards the subsets of patients in whom benefit is gained but not recognized, or in those who are harmed overtly or covertly but in whom clinicians continue to use the intervention. Individual benefit and detriment are subsumed within neutral overall population outcomes. The belief that these concerns could be overcome by enrolling thousands, if not tens of thousands, of patients into large-scale pragmatic studies, has not helped, as the lack of consistent effect will be unaffected by the size of the population.

It should be emphasised that some interventions can be reasonably assumed to not offer any outcome or cost benefit and can be abandoned, especially if consistently neutral or harmful findings are generated from multiple RCTs. A good example is the use of hetastarches for fluid resuscitation. Compared against crystalloid solutions in both non-selected critically ill patients, as well as in relevant patient subsets such as sepsis, shock and trauma, there was benefit from hetastarches in terms of reduced volume requirements, but an increased risk of mortality and renal replacement therapy [5]. Intriguingly, a similar meta-analysis performed in surgical patients confirmed the haemodynamic benefits of hetastarches over crystalloids, including a reduced need for vasopressors, but with no impact on renal function or mortality, and a decrease in length of hospital stay [6].

The converse scenario also applies. For example, the RECOVERY studies reported survival benefit from corticosteroids and interleukin-6 blocking agents in COVID-19 disease [7, 8]. Multiple issues with the design and conduct of these highly pragmatic trials have since been highlighted [9]. Notably, the benefit could not be replicated in Cochrane reviews which concluded there was little or no clinical improvement from these drugs at one month [10, 11].

### Examples of large pragmatic ICU trials with reasons for inconclusive results

Depending on the intervention under investigation and/or the study design, neutral outcome pragmatic trials may result from overly broad patient selection, protocol non-adherence, or use of fixed dosing regimens or inappropriate targets that may either under- or over-treat the condition. We have selected some examples below, not that they were any more egregious than other pragmatic studies, but on the basis they were published in high impact factor journals, are well-known and highly cited.

#### (i) Mean arterial pressure targeting in vasodilatory hypotension/septic shock

**Fig. 3:**
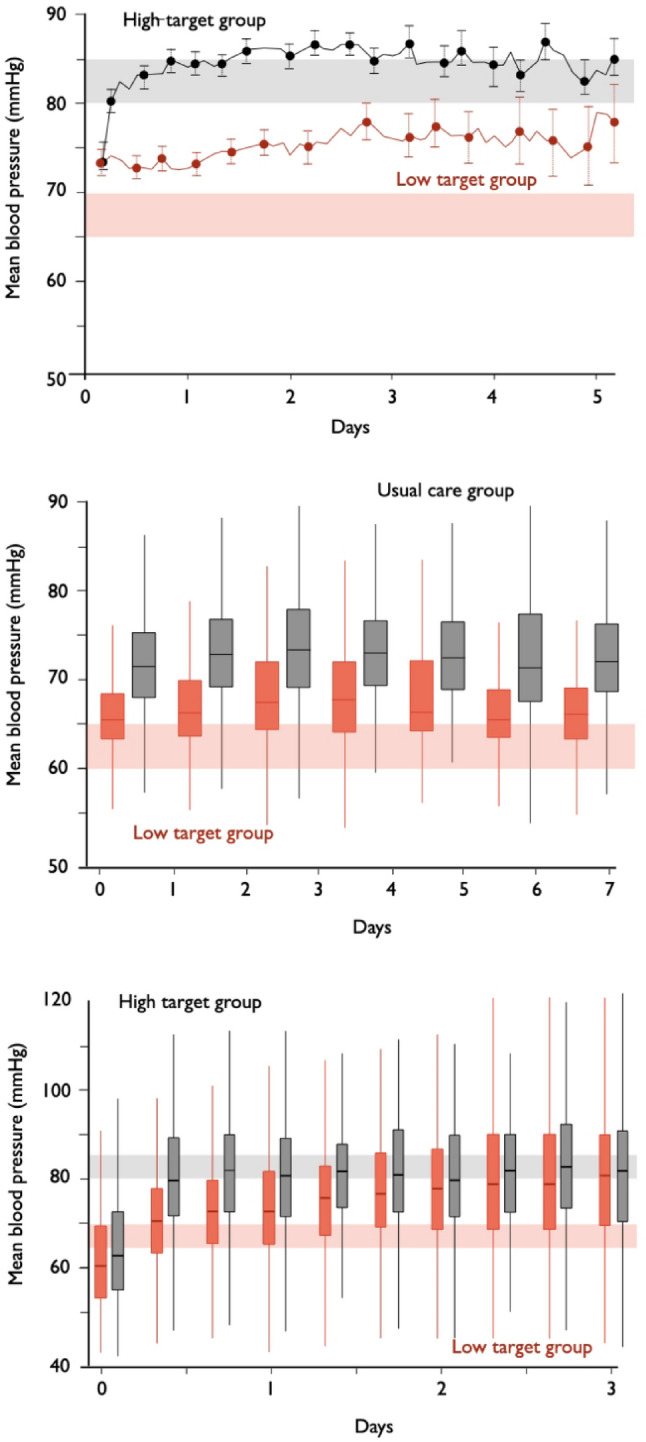
Mean arterial pressure values in the SEPSISPAM (top panel), 65 (middle panel), OPTPRESS (lower panel) randomised control trials (adapted from Refs [12-14]). Values are shown as median and 95% confidence intervals (**a**), median, quartile and 1.5x inter-quartile range (**b**) and median, quartile and unstated (**c**).

SEPSISPAM, 65 and OPTPRESS were three large pragmatic RCTs performed in France, UK and Japan, respectively, to assess ‘optimal arterial pressure’.^12–14^ Each aimed to identify an optimal mean arterial pressure target for patients with septic shock, plus other causes of vasodilatory hypotension in the 65 study. The 65 and OPTPRESS trials only included patients ≥ 65 years. The mean arterial pressure (MAP) targets were 65–70 (low) and 80–85 (high) mmHg for both SEPSISPAM and OPTPRESS, and 60–65 mmHg vs. ‘usual care’ for the 65 trial. OPTPRESS was discontinued prematurely because of an excess mortality signal in the high MAP target group, although this was not seen in the other two studies (Supplementary Table 1). A possible explanation is an excess use of vasopressors, especially vasopressin, in the higher target group in OPTPRESS [15]. Notably, none of the trials came close to achieving the MAP target goals over the study period (3–7 days), let alone Day 1. In addition, marked variability in MAP values occurred at each timepoint in 65 and OPTPRESS; only SEPSISPAM achieved clear between-group separation (Fig. 3).

So what conclusions, if any, could be drawn from these studies, enrolling nearly 4000 patients, that could inform guidelines and routine practice? The pragmatic design of the trials, with usual bedside nursing and junior medical staff being responsible for continuous titration of vasopressors to the target arterial pressure range, clearly failed to follow the protocol in the majority of patients. This created excessive variation in treatment targets, overlap between treatment groups, generating uninterpretable data and null treatment effect inferences.

#### (ii) Albumin

**Fig. 4:**
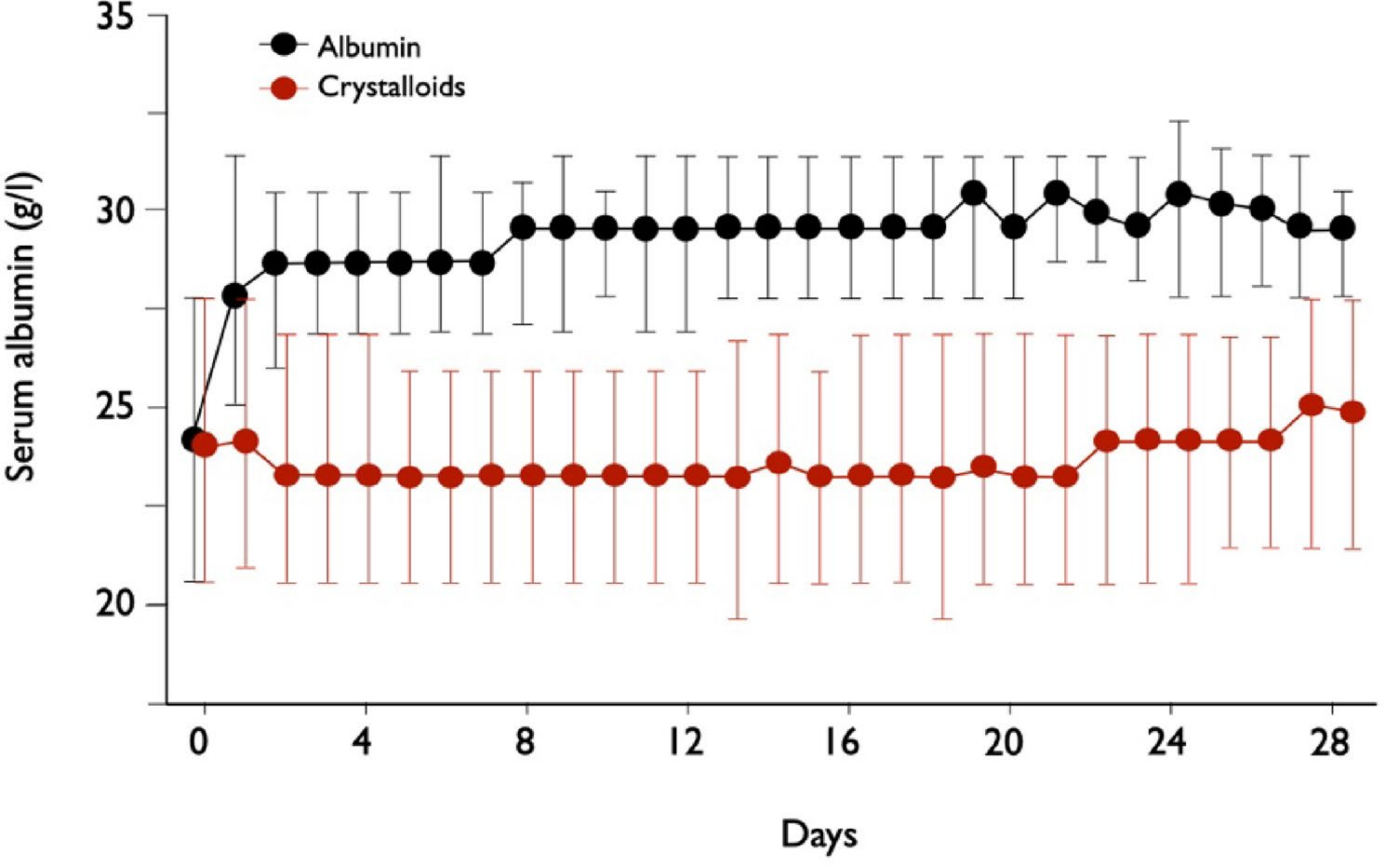
Serum albumin levels over 28 days in the ALBIOS study (adapted from Ref [16]). Data shown as median and interquartile range

The ALBIOS multicentre, open-label trial enrolled 1818 patients with sepsis who were randomly assigned to receive crystalloid solution with or without 20% albumin [16]. In the albumin treatment group, the target serum albumin concentration was ≥ 30 g/L until ICU discharge or 28 days after randomization. No difference between groups was seen in either the primary outcome (28-day mortality) nor any secondary outcome. While there was separation between the groups, less than half the patients in the albumin group achieved the 30 g/L target (Fig. 4), yet albumin administration from days 2–7 rarely exceeded 200 ml/day after Day 1 (Supplementary Table 2).

The underlying rationale for ALBIOS was that raising albumin would have beneficial effects on plasma colloid osmotic pressure, offer antioxidant and anti-inflammatory properties, and aid acid–base buffering. Did the failure to meet the pre-defined target level compromise the chances of success?

### Looking forward

The protagonists of pragmatic trials argue that the design and conduct of such studies reflects the use of the intervention in real world practice. If it does not work, the intervention can be discarded, saving money and effort, and we can then move on to the next large pragmatic trial. Surely, the emphasis should be on improving the conduct of the trial to give the intervention the best chance of showing benefit (or harm), and to identify in whom these apply. If so, concerted efforts should then be made to improve real world practice to perform the intervention correctly.

The emphasis of pragmatic ICU trials has traditionally been placed on the primary outcome, however the result has rarely been anything other than neutral. The biological rationale underpinning a pragmatic trial must be carefully considered. Was the concept of the three MAP target trials [12–14] described above fundamentally flawed? Would throwing disparate patients together – young and old, hypertensive and normotensive - into a one-size-fits-all strategy be expected to yield a clear primary outcome result? Such trials largely ignore the heterogeneity of the ICU population where a proportion would perhaps benefit from the intervention, a proportion may be harmed, and the remainder would be unaffected. Lack of generalisability to all patients, a criticism against explanatory studies, should not be used to preclude their use in specified populations. This message is being embraced in more recent studies in sepsis and ARDS such as ImmunoSep [17] and PANTHER [18] where immunomodulatory treatments are being targeted to specific endotypes. Indeed, a strong argument could be made to jettison the long-established concept of syndromic trial enrolment as no longer fit-for-purpose. Pragmatic trials could also be enhanced by more detailed explanatory analyses in a subset of patients, for example blood sampling, to better understand why patients respond positively or adversely.

Subgroup analyses from large pragmatic trials are thus relevant but underexplored. Such analyses may be underpowered but may show a positive result through enrichment. Necessary caveats include the fulfilment of important criteria such as pre-specification and biological plausibility, plus evidence of interaction and consistency across related outcomes. Caution should be applied to over-interpretation of secondary findings, especially from post hoc analyses, with generatin of excessive clinical claims. These data do allow hypothesis generation, enabling follow-on confirmatory studies focussed on specific target groups. Again, these analyses are contingent on studies that avoid a high proportion of protocol violations to accurately assess the intervention’s impact.

An unintended consequence of the limitations in trial design and conduct is their impact on meta-analyses and management guidelines. Clinical diversity, intervention heterogeneity, variable protocol adherence, different comparator strategies, and inconsistent outcome definitions can easily compromise pooled average effects. Structured assessments of between-trial clinical diversity accompany assessments of statistical heterogeneity [19]. The variable findings from multiple studies comparing high versus low levels of positive end-expiratory pressure (PEEP) (Table 2) [20–23] prompted a Cochrane review [24] to conclude “*clinical heterogeneity - mainly within participant characteristics and methods of titrating PEEP ‐ does not allow us to draw definitive conclusions regarding the use of high levels of PEEP in patients with ALI and ARDS. Further studies should aim to determine the appropriate method of using high levels of PEEP and the advantages and disadvantages associated with high levels of PEEP in different ARDS and ALI patient populations*.”

**Table 2:** Disparity in mortality across the major randomised controlled studies comparing high vs. low positive end-expiratory pressure Set PEEP (cm H_2_O) on Day 1

Set PEEP (cm H_2_O) on Day 1			
		Low PEEP	High PEEP
ALVEOLI	Mean (SD)	8.9 (3.5)	14.7 (3.5)
LOV	Mean (SD)	10.1 (3.0)	15.6 (3.9)
ExPress	Mean (SD)	7.1 (1.8)	14.6 (3.2)
ART	(95% CI)	12.0 (11.7–12.3)	16.2 (15.9–16.6)

**Mortality (%)**			
	Mortality	Low PEEP	High PEEP
ALVEOLI	Hospital	24.9	27.5
LOV	28-day	32.3	28.4
ExPress	28-day	31.2	27.8
ART	28-day	49.3	55.3

Training and study monitoring should be enhanced to improve protocol compliance. As highlighted by the examples above, bedside providers are likely less invested and trained than the named study site investigators with, perhaps, insufficiently rigorous bedside supervision. This issue is commonplace. Poole et al. conducted an individual patient data analysis of tidal volumes used in three UK pragmatic multicentre RCTs of ARDS and found poor compliance (only 20–39%) with the well-established 6–8 ml/kg recommendation for tidal volume [25]. Training should extend to all non-research clinical staff involved in performance of the study, highlighting the potential importance of the study result with regular reinforcement and encouragement, and close engagement in the study from medical and nursing leadership. Trial monitoring should extend beyond patient recruitment and accurate completion of the case record forms to scrutinising adherence to the protocol with regular feedback to study sites. This could include real-time monitoring and notification of protocol deviations, automated alerts, dashboard-based feedback and site-level performance reports.

Per-protocol analysis is performed on data from patients in whom there are no major pre-defined protocol violations and the pre-specified intervention has been completed. This approach aims to confirm treatment effects under optimal conditions. However, as this analysis excludes non-adherent patients, it has been frowned upon in statistical circles as it can potentially introduce selection bias and confounding. The balance usually achieved between groups by the initial randomization may be lost, and patients (or ICUs) where treatment is more protocol-adherent may differ in ways that influence their outcomes [26]. Nonetheless, as noted by Smith and colleagues [27]: “*Both the estimated effect of being assigned to a treatment (from an intention-to-treat (ITT)-based analysis) and the estimated effect of full adherence with a treatment may be useful to both clinicians and patients to fully understand the range of potential treatment effects. The treatment effect estimated from per-protocol or as-treated analyses is frequently larger than the effect size estimated from the ITT-based approach*,* particularly when adherence to treatment is associated with larger treatment effects.”*

Per-protocol analysis should not be viewed in isolation but it does provide added confidence in the ITT results of a trial, or can at least hypothesis generate to indicate a possible benefit from following the protocol. An alternative (or additional) option is to use casual inference methods. The BALANCE trial is an excellent recent example [28]. This large multicentre, multinational noninferiority trial of 3608 patients compared antibiotic treatment for 7 or 14 days in patients with bloodstream infection. An advantage of their study design was the required inclusion criteria of patients with microbiological confirmation of bloodstream infection and antibiotic sensitivities, and a pre-specified exclusion of patients with likely contaminated cultures, thus avoiding enrolment of non-infected patients. The ITT analysis showed non-inferiority for the primary outcome (death from any cause at 90 days), all secondary outcomes and across prespecified subgroups related to patient, pathogen and syndrome characteristics. Noninferiority was preserved in a modified ITT analysis excluding patients who died before day 7 of treatment.

The investigators did however note that 23.9% of patients in the 7-day group and 16.5% of patients in the 14-day group were treated for ≥ 2 days shorter or longer than the assigned duration. A per-protocol analysis was accordingly performed and this confirmed noninferiority between the two treatment approaches. An intriguing post-hoc analysis using causal inference methods, including inverse probability of treatment weighting (IPTW) and instrumental variable methods, was performed to identify factors associated with this high non-adherence (overall 20.3%), and any possible effect on the validity of the trial results [29]. They found disease severity, intra-abdominal, skin or soft tissue sources of infection, persisting pyrexia and persistent bacteraemia were associated with longer treatments, while a vascular catheter source of infection and antimicrobial resistance were associated with shortened treatment. The authors reported a consistent conclusion of non-inferiority, strengthening the validity of the initial ITT analysis and their recommendation that 7-day antibiotic duration should be the standard of care for patients with uncomplicated bloodstream infection.

Another approach to consider is assessment of individual treatment effects. This has recently been applied to the early goal-directed therapy (EGDT) studies. To refresh memories, Rivers and colleagues conducted an open-label single-centre RCT of 263 septic patients presenting to the Emergency Department, comparing standard-of-care management against a specified strategy of fluid resuscitation ± blood transfusion ± dobutamine with a target central venous saturation (ScvO_2_) ≥ 70% over the first 6 h from randomization [30]. MAP and central venous pressure targets were similar. A significant reduction in in-hospital mortality (30.5% vs. 46.5%) was seen in the EGDT group compared to the standard-of-care group. The EGDT concept was subsequently endorsed and heavily promoted by the Surviving Sepsis Campaign. However, no outcome benefit was seen with EGDT when the strategy was replicated in three pragmatic multicentre effectiveness studies performed in the USA (ProCESS), Australia (ARISE) and the UK (ProMISe) [31–33]. A subsequent individual patient data meta-analysis (IPDMA) of the three EGDT trials failed to identify any subgroups that benefitted from EGDT. [34]. Only intention-to-treat analyses were performed within the three trials. However, in ARISE and ProMISe 20–30% of patients in the EGDT group did not achieve the ScvO_2_ target of 70% while ProCESS did not report compliance rates and, perhaps surprisingly, accepted rather wide ranges of ScvO_2_ and MAP as protocol-adherent.

Shah et al. recently re-analysed the ProCESS and ARISE studies [35]. They hypothesised that the average treatment effect (ATE) would not reflect an individual treatment effect (ITE), especially with the wide variations in fluid and vasopressors administered. Thus, a possible heterogeneity of treatment effect (HTE) had passed unidentified by conventional statistics. They modelled treatment response at an individual patient level and ranked these in quintiles according to likelihood of benefit. Notably, the response to EGDT ranged from harm in the first quintile (31% vs. 23% mortality in the usual care group), to benefit in the fifth quintile (21% vs. 31% mortality). Compared to the fifth quintile, patients in the first quintile were younger, more tachycardic, with a higher plasma bilirubin, and lower values of albumin, urea, creatinine, glucose, platelet and white cell count. Clearly, these findings are modelled on available retrospective data, and would need prospective validation. It is nonetheless intriguing that patient characteristics, predominantly biochemical covariates, could differentiate positive and negative responders to the same intervention within a pragmatic trial, albeit no HTE could be identified among the individual EGDT components.

Similar exercises have been applied to other interventions where an overall outcome benefit has not been shown. Pirrachio et al. modelled data from 2548 septic patients enrolled into four multicentre RCTs and concluded that the ITE of corticosteroid treatment did yield a positive net benefit [36]. Zampieri and colleagues estimated a conditional ATE in 10,465 critically ill patients enrolled into the BaSICS trial comparing fast (999 ml/h) vs. slow (333 ml/h) fluid infusion rates [37]. In 19% of patients a recommendation could be made, with slow infusion more often recommended in younger patients having planned ICU admissions, and fast infusion in older patients with sepsis. These recommendations were associated with an odds ratio for hospital mortality of 0.58 (95% credible interval 0.32–0.90; 0.99 posterior probability of benefit) and 0.72 (credible interval 0.54–0.91; probability of benefit > 0.99), respectively.

A feature of many pragmatic trials is the relative paucity of data collected. While such a trial may inform on the overall performance of the intervention, it may then prove difficult to identify specific components or biases that explain the obtained result. Furthermore, sparse data will compromise the ability to perform an individual treatment effect analysis. With increasing availability of electronic healthcare records (EHRs), it is conceivable that these comprehensive datasets, or at least selected, non-identifiable data extracted from them, could be integrated into the study database, acknowledging the medicolegal and technical challenges involved. Machine learning approaches can then be applied to aid identification of likely intervention responders.

Newer trial designs carry potential advantages but also new challenges. Adaptive platform trials offer the possibilities of adjusting to response-adaptive features and prospectively testing treatment-effect heterogeneity [38], while Bayesian approaches could facilitate exploration of subgroup effects and simulation of operating characteristics. Yet caution needs to be applied, for instance, ensuring transparent specification of priors and not overinterpreting probabilistic subgroup signals.

## Summary

Explanatory trials evaluate the efficacy of an intervention under optimal conditions and in well-defined populations, aiming to minimise bias and confounding and to maximise the treatment effect. By contrast, pragmatic trials test the effectiveness of the intervention within real-life clinical practice, prioritising applicability and generalisability. There are no truly pragmatic or explanatory trials; some are designed to have aspects (domains) that are more pragmatic than explanatory, and vice versa. Some trials should be more explanatory to provide high internal validity for new interventions and identification of cause-effect relationships. Pragmatic trials may generate post-hoc exploratory analyses which will, in turn, require verifying explanatory trials.

A major challenge in a highly heterogenous ICU population is to distinguish responders from non-responders to an intervention [39]. Broad inclusion of both patient subtypes, with a subsequent inability to separate them, has contributed to many pragmatic trials with neutral outcomes. This has neither advanced knowledge nor improved patient management. The sometimes suboptimal design and/or conduct of pragmatic ICU studies, where protocol non-adherence is often excessive, may reflect real-life practice but endangers throwing the baby out with the bathwater; potentially useful interventions in some patient subsets are abandoned when effectiveness cannot be demonstrated. The onus is on trial investigators to improve study designs, including realistic recruitment rates and proposed treatment effects, and to better inform, train and motivate clinical staff to follow the protocol.

Per-protocol analyses, with careful consideration of potential bias and confounding, may be useful to confirm or refute the ITT analysis. Post hoc causal inference techniques with individual treatment effects may also identify patient groups likely to benefit or not. The increasing availability of high granularity electronic data, allied to ever more sophisticated machine learning techniques, may enhance interpretation and application of pragmatic trials.

Taken together, exploratory, *post hoc*, subgroup, per-protocol, as-treated, causal inference, and machine-learning-derived findings are useful for hypothesis generation, trial refinement, and mechanistic understanding. However, these should not be considered equivalent to confirmatory evidence. Findings derived from such methods should generally be corroborated in subsequent trials with prospective hypotheses, adequate power, robust intervention separation, and high standards of trial conduct and adherence.

## Supplementary Material

Below is the link to the electronic supplementary material.

Supplementary Material 1.

## Data Availability

No datasets were generated or analysed during the current study.
