# Supplementary material for: What place for pragmatic RCTs in intensive care?
Source: Crit Care. 2026 Aug 12;30:411. doi: 10.1186/s13054-026-06242-z (PMC13464313; doi:10.1186/s13054-026-06242-z)
Supplement: Supplementary file 1 [file 13054_2026_6242_MOESM1_ESM.docx]

**Supplementary Table 1: Outcome data from three randomised controlled trials (SEPSISPAM, 65, OPTPRESS)^12-14^ studying mean arterial pressure targets in patients with shock.**

* p<0.05

|  | SEPSISPAM | | 65 | | OPTPRESS | |
| --- | --- | --- | --- | --- | --- | --- |
| Number enrolled | 776 | | 2463 | | 518 | |
| Mean arterial pressure target (mmHg) | 65-70 | 80-85 | 60-65 | ‘usual care’ | 65-70 | 80-85 |
| Outcome   - 28 day mortality - ICU mortality - 90 day mortality | 34%  -  42.3% | 36.6%  -  43.7% | 29.9%  41% | 30.7%  43.8% | 21.6%  -  28.6% | 30.4%*  -  39.3%* |
| Chronic hypertension subgroup  -need for RRT  -RRT-free days at Day 28  -90 day mortality | 42.2%  -  - | 31.7%*  -  - | 28.3%  -  - | 24.6%  -  - | -  19  31.8% | -  18  38.1% |
| Normotension subgroup  -need for RRT  -RRT-free days at Day 28  - 90 day mortality | 30.7%  -  - | 34.8%  -  - | 21.8%  -  - | 24.7%  -  - | -  22  25.4% | -  17*  40.5% |

RRT renal replacement therapy

**Supplementary Table 2: Albumin dosage given per day to albumin treatment group over the first 7 days in the ALBIOS study (from Reference 16).**

Data shown as median and interquartile range

| Day | No of patients | Albumin given (ml) |
| --- | --- | --- |
| 1 | 841 | 300 [300-300] |
| 2 | 789 | 200 [0-200] |
| 3 | 742 | 200 [0-200] |
| 4 | 702 | 200 [0-200] |
| 5 | 639 | 200 [0-200] |
| 6 | 586 | 200 [0-200] |
| 7 | 542 | 100 [0-200] |
